# Characteristics of patients with missing information on stage: a population-based study of patients diagnosed with colon, lung or breast cancer in England in 2013

**DOI:** 10.1186/s12885-018-4417-3

**Published:** 2018-05-02

**Authors:** Chiara Di Girolamo, Sarah Walters, Sara Benitez Majano, Bernard Rachet, Michel P. Coleman, Edmund Njeru Njagi, Melanie Morris

**Affiliations:** 1 Cancer Survival Group, Faculty of Epidemiology and Population Health, Department of Non-Communicable Disease Epidemiology, London School of Hygiene & Tropical Medicine, Keppel St, London, WC1E 7HT UK; 2 Department of Medical and Surgical Sciences, Alma Mater Studiorum – University of Bologna, Via Zamboni, 33 40126 Bologna, Italy

**Keywords:** Cancer, Neoplasm, Population-based, Stage, Missing data, England

## Abstract

**Background:**

Stage is a key predictor of cancer survival. Complete cancer staging is vital for understanding outcomes at population level and monitoring the efficacy of early diagnosis initiatives. Cancer registries usually collect details of the disease extent but staging information may be missing because a stage was never assigned to a patient or because it was not included in cancer registration records. Missing stage information introduce methodological difficulties for analysis and interpretation of results. We describe the associations between missing stage and socio-demographic and clinical characteristics of patients diagnosed with colon, lung or breast cancer in England in 2013. We assess how these associations change when completeness is high, and administrative issues are assumed to be minimal. We estimate the amount of avoidable missing stage data if high levels of completeness reached by some Clinical Commissioning Groups (CCGs), were achieved nationally.

**Methods:**

Individual cancer records were retrieved from the National Cancer Registration and linked to the Routes to Diagnosis and Hospital Episode Statistics datasets to obtain additional clinical information. We used multivariable beta binomial regression models to estimate the strength of the association between socio-demographic and clinical characteristics of patients and missing stage and to derive the amount of avoidable missing stage.

**Results:**

Multivariable modelling showed that old age was associated with missing stage irrespective of the cancer site and independent of comorbidity score, short-term mortality and patient characteristics. This remained true for patients in the CCGs with high completeness. Applying the results from these CCGs to the whole cohort showed that approximately 70% of missing stage information was potentially avoidable.

**Conclusions:**

Missing stage was more frequent in older patients, including those residing in CCGs with high completeness. This disadvantage for older patients was not explained fully by the presence of comorbidity. A substantial gain in completeness could have been achieved if administrative practices were improved to the level of the highest performing areas. Reasons for missing stage information should be carefully assessed before any study, and potential distortions introduced by how missing stage is handled should be considered in order to draw the most correct inference from available statistics.

## Background

Stage at diagnosis is a key predictor of cancer survival: a higher stage is associated with lower survival. Besides its relevance for the clinical management of individual patients, good quality and complete cancer staging is vital for understanding outcomes at population level and monitoring the efficacy of early diagnosis initiatives [1–3].

Tumour staging is usually done through clinical investigations, such as imaging, and/or diagnostic or surgical procedures aimed at determining the pathological extension of the tumour.

The Tumour Node Metastasis (TNM) classification [4], maintained and periodically updated by the International Union for Cancer Control (UICC), is an international standard for determining disease extension and, ultimately, treatment options. It is based on the combination of three components: size and extent of the primary tumour (T), nodal involvement (N) and presence or absence of distant metastasis (M). Population-based cancer registries usually collect these details of the disease extent to derive a TNM stage grouping, however the completeness of this stage information may vary by cancer site and by registry.

Missing stage information introduces methodological difficulties for analysis, and for interpretation of results. Understanding the characteristics of patients with missing stage and the mechanisms behind missing data is crucial to evaluating the potential extent of bias and to reaching sensible conclusions.

Stage information may be missing either because it was never assigned or because it was not documented in cancer registration records. In the first case, the lack of stage may result from an incomplete staging assessment that in turn may be associated with socio-demographic and clinical characteristics of the patient. Previous research carried out in England, Europe, and the United States has reported that the proportion of unstaged cancers is higher in certain subgroups of the population such as the elderly [5–8], those with high levels of comorbidity or complex care needs, and those in institutionalised settings [9–12]. Race, gender, marital status, place of residence, and receipt of surgical treatment have also been associated with missing stage [5, 13, 14]. Patients’ refusals to undergo staging investigation procedures may also contribute to missing information. In certain settings such as England, patients diagnosed and/or treated exclusively in private hospitals are also less likely to have their data captured by the national registry system. Under all of these scenarios, stage information is plausibly missing at random (MAR): that is, the probability for the stage to be missing is not related to the stage itself (e.g. advanced stage), but conditional on other characteristics of the patients (e.g. age, comorbidity, deprivation) [15]. Instead, if the only cause for missing stage were the presence of obvious metastatic disease or direct referral to palliative care, it would be more likely that stage data are missing not at random (MNAR). This means that, even after accounting for observed patients’ characteristics, there will still be systematic differences in the stage distribution between those with complete and missing stage information.

In some instances, although stage may have been known to the clinician or recorded in the clinical notes, it was not reported to the cancer registry. Errors in coding of the data or failures in the recording system can contribute to lack of stage reporting. When administrative or communication issues are the cause of missing data, the stage information is likely to be missing completely at random (MCAR) at regional and national level, meaning that the missing data can be considered to be a random subset of the data. In these cases, the exclusion of the records with missing values from the analysis (complete case analysis) would still  produce unbiased estimates, but  it  would lead to a loss of statistical power and to a corresponding increase in variance.

Improving stage completeness has been a priority in recent years in England, with financial and human resources being invested to increase stage reporting to the cancer registry. Actions have included the improvement of the IT systems at hospitals and registries, the assignment of data liaison teams to go into hospitals to investigate and improve data collection practices, and the introduction of standardised collection and recording procedures. As an illustration, completeness of stage was about 70% for patients diagnosed with non-small cell lung cancer between 2004 and 2007 [16]. In 2012, stage completeness reached 80–90% for patients diagnosed with one of the four major cancers (colorectal, lung, breast, and prostate) [17]. Since 2014, the percentage of newly diagnosed cancers recorded with a valid stage has been a quality indicator for Clinical Commissioning Groups (CCGs), the National Health Service (NHS) units in which cancer services are budgeted and planned in England [18]. Although CCGs are not directly responsible for collecting cancer data, they can be considered as proxies for the registration practices of the hospital trusts in the areas for which they commission services.

Despite recent improvements, there remains a proportion of patients for whom there is no valid stage information in the cancer registration data. In this study, we aim to describe the socio-demographic and clinical characteristics of patients diagnosed with colon cancer, non-small cell lung cancer or breast cancer in England in 2013, for whom stage information was not available in the cancer registration data. We describe geographical patterns of completeness, and investigate whether the association between patients’ characteristics and missing stage information differs when completeness is high. We also estimate the number of patients who might have been assigned a stage if the highest levels of completeness were achieved throughout the country. Finally, we use multiple imputation to estimate the likely stage distribution of patients with missing stage.

## Methods

All patients aged 15–99 years diagnosed with a primary, invasive, malignant colon cancer (International Classification of Diseases, ICD-10 C18), non-small cell lung cancer (ICD-10 C34, lung cancer hereafter), and all women diagnosed with breast cancer (ICD-10 C50) in England in 2013 were identified in the national cancer registration system [19]. These three cancer sites were chosen as they are among the most common malignancies, but have different prognosis, age and stage distributions at presentation.

### Data sources and specification

Individual cancer records were retrieved from the National Cancer Registration and Analysis Service (NCRAS) through the Cancer Analysis System (CAS). This database collates multiple cancer sources and contains various pieces of information about the tumour stage. In the present study, we used the ‘registry-derived stage’, a TNM stage grouping variable derived by NCRAS using internal procedures from available pathological and clinical information. This variable is generally used in institutional reporting in England. Whether or not a patient had a valid registry-derived stage in their record was the outcome of interest.

National cancer registration data in England include quality-checked information on each patient’s date of birth, gender, vital status, follow-up dates, tumour morphology, the former regional registry under which the cancer case was registered (the eight former regional registries merged into a single national one in 2013), and the postcode of residence at the time of diagnosis. Postcode was used to map patients to a small geographical region, the Lower layer Super Output Area (LSOA), and to assign them to a CCG and to one of the five ecological deprivation quintiles, derived from the income domain of the Index of Multiple Deprivation for England [20]. The least deprived quintile is coded to 1, and the most deprived to 5. As there were no clear or consistent gradients across these five categories and numbers in some categories were small in some CCGs, we further grouped patients into two categories: more affluent (quintiles 1 and 2), and more deprived (quintiles 3, 4 and 5). We derived a variable as an indicator of short-term mortality based on the vital status of each patient at 30 days after diagnosis.

Individual tumour observations were linked to the Routes to Diagnosis (RtD) dataset [21] in order to classify patients into two groups according to whether they were diagnosed through an emergency admission or not, and to the Hospital Episode Statistics (HES) dataset in order to acquire additional clinical information. We applied an algorithm developed by Maringe et al. [22] to the HES dataset to obtain information on prevalent comorbidities diagnosed up to six years before the cancer diagnosis (excluding the six  months preceding the cancer diagnosis as they might have been a consequence of the tumour itself) which were then summarised into the Charlson Comorbidity Index (CCI). This index assigns patients an overall score composed of a sum of severity-weighted values given to 17 specific chronic conditions [23]. We defined three levels of comorbidity according to the overall score (low = 0; medium = 1–2; high ≥3). Patients were classified as having received surgery if they were recorded in HES as having undergone a cancer-related procedure up to six months after the cancer diagnosis.

### Statistical analyses

In the descriptive analyses, a chi-squared test was used to test for significant differences between patients with and without stage information for each of the independent variables.

One-year stage-specific net survival was calculated using the Pohar-Perme estimator, which accounts for competing risks of death from other causes with increasing age via inverse probability weighting [24], using the standard cohort approach and follow-up to 31/12/2014 [25]. Mortality from other causes by age and sex was drawn from region- and deprivation-specific life tables for England for 2013 and 2014 [26]. An additional missing stage category was included in the stratified analysis. In order to enable comparison between stages, estimates were age-standardised using the International Cancer Survival Standards (ICSS) weights, for which age at diagnosis was categorised into five groups (15–44, 45–54, 55–64, 65–74, and 75–99 years) [27].

In order to account for the potential variability in the outcome between the 209 CCGs, we used multivariable beta binomial regression models [28, 29] to estimate the odds ratios (OR) for the association between socio-demographic and clinical characteristics of patients and missing stage. Two sets of models, each containing all the socio-demographic and clinical variables available, were built. Firstly, models included all patients diagnosed in 2013 in order to understand what factors were associated with missing stage (Model 1). Secondly, analyses were restricted to CCGs with a low proportion of missing stage data amongst their patients, to allow further investigation of which patient factors remained associated with missing stage in areas with high completeness (Model 2). In these areas we assumed that patient characteristics were the main determinants of missing data, while administrative or communication issues played a marginal role. These were the CCGs for which the percentage of missing stage data was below the 10th percentile of the distribution across all CCGs (8.2% for colon, 5.2% for lung, and 4.7% for breast cancer). We tested for interactions between various patient characteristics and we did not find any statistically significant interaction apart from an effect modification between age and comorbidities among women diagnosed with breast cancer. Since this interaction only slightly changed the estimates and was non-significant in the model restricted to CCGs with high completeness (Model 2), we only reported the results from the main models (without interactions).

A question of interest was whether the proportion of missing stage would have changed if the missingness patterns of the areas with high completeness were applied. To achieve that, the parameter estimates from the Model 2 for the restricted sample of CCGs with high completeness of stage data were applied to the various covariate patterns on the full cohort in order to calculate the number of patients who might have been expected to have valid data on stage. The expected number of patients with missing stage data was then subtracted from the observed number to provide an estimate of the number and proportion of patients for whom missing data on stage was potentially avoidable, if they had had the same chance of being staged as the patients in CCGs with high completeness where administrative issues were assumed to be minimal.

Under the assumption that stage was missing at random, we estimated the likely stage distribution of patients with missing stage using multiple imputation. As the proportion of missing stage was never above 20%, 20 imputed datasets for each cancer site were produced using the *mi impute* Stata command and a multinomial logistic modelling approach that included all the variables used in the analysis as well as the morphological subtype (for breast cancer), the former regional cancer registry, the event indicator and the Nelson-Aalen estimator of the cumulative hazard of death [15]. Analyses were conducted using Stata 14 [30].

## Results

The study population consisted of 21,522 colon cancer patients, 31,188 lung cancer patients, and 41,657 women with breast cancer diagnosed in England in 2013. The proportion of patients with missing stage information was 18.5% for colon, 12.6% for lung and 15.6% for breast cancer.

For each cancer, data on stage were more often missing for older patients (youngest versus oldest age group: 15% vs 32% for colon, 10% vs 22% for lung, 13% vs 38% for breast cancer), for those who were diagnosed through an emergency route, who died within 30 days of diagnosis (patients who survived more than 30 days versus patients who died within 30 days: 17% vs 37% for colon, 10% vs 25% for lung, 15% vs 48% for breast cancer), those who did not have record of cancer-directed surgery, or who had concomitant chronic conditions (Table 1). For patients with cancers of the lung or breast, the proportion with missing stage was significantly higher among more deprived patients. For colon cancer, the proportion was higher among women than men.

**Table 1:** Distribution of patients with missing stage information, and net survival by stage, England, 2013

	Colon cancer			Non-small cell lung cancer			Breast cancer		
	No.	Missing stage (%)		No.	Missing stage (%)		No.	Missing stage (%)	
All patients	21,522	18.5		31,188	12.6		41,657	15.6	
Age at diagnosis									
15–64	5566	15.1	*	6890	9.7	*	22,611	12.7	*
65–74	6024	14.5		10,161	9.9		9376	11.5	
75–84	6798	18.6		10,008	13.4		6393	20.4	
85+	3134	32.1		4129	22.4		3277	38.3	
Sex									
Male	11,316	17.6	*	17,004	12.3				
Female	10,206	19.6		14,184	13.0				
Income deprivation group									
More affluent	9489	18.7		9769	13.5	*	18,771	16.9	*
More deprived	12,033	18.4		21,419	12.2		22,886	14.6	
Emergency presentation									
No	15,248	17.3	*	20,549	10.8	*	40,137	15.1	*
Yes	6274	21.5		10,639	16.0		1520	31.1	
Death within 30 days of diagnosis									
No	19,973	17.1	*	25,919	10.1	*	41,273	15.3	*
Yes	1549	36.5		5269	24.8		384	48.4	
Surgical procedure									
No	3403	35.7	*	26,410	13.7	*	9450	37.4	*
Yes	18,119	15.3		4778	6.8		32,207	9.3	
Charlson comorbidity index									
0	15,913	17.1	*	19,398	12.1	*	34,782	14.6	*
1–2	4009	21.3		8478	12.7		5376	18.9	
> =3	1600	25.6		3312	15.7		1499	28.2	
One-year net survival (NS %, 95% CI)									
Stage I	0.98	(0.98–0.99)		0.88	(0.87–0.89)		1.00	(1.00–1.01)	
Stage II	0.95	(0.94–0.96)		0.73	(0.71–0.75)		1.00	(0.99–1.00)	
Stage III	0.89	(0.89–0.90)		0.48	(0.47–0.50)		0.96	(0.95–0.97)	
Stage IV	0.44	(0.42–0.45)		0.20	(0.19–0.20)		0.67	(0.65–0.67)	
Missing stage	0.72	(0.70–0.74)		0.36	(0.34–0.37)		0.92	(0.92–0.93)	

^*^p-value from chi-squared test ≤0.05

Age-standardised one-year net survival among patients without a valid stage was 72% for colon, 36% for lung and 92% for breast cancer (Table 1). For colon and lung cancer patients, survival estimates of these were between those of patients diagnosed with stage III and stage IV tumours. Survival of women diagnosed with breast cancer and with missing stage was closer to that seen in patients with stage III breast cancer.

**Fig. 1:**
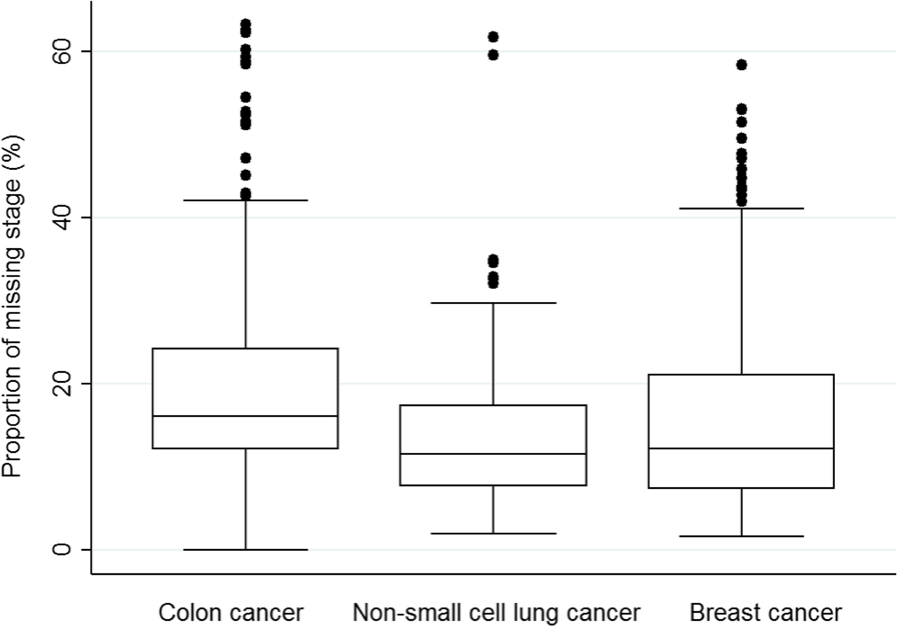
Distribution of the proportion of missing stage by Clinical Commissioning Group, England, 2013

There was substantial variation in the proportion of patients with missing stage between the 209 CCGs: the range was 0–63% for colon, 2–62% for lung, and 2–58% for breast cancer (Fig. 1).

For each cancer site, findings for the whole cohort (Model 1, point estimates mutually adjusted for all the other covariates) indicate that increasing age was associated with missing stage information: patients aged 85 or older had approximately twice the odds of missing stage compared with patients aged 15–64 (colon cancer: OR = 1.8, 95%CI 1.6–2.1; lung cancer: OR = 2.1, 95%CI 1.4–2.4; breast cancer: OR = 2.2, 95%CI 1.8–2.6). Additionally, patients who died shortly after diagnosis and those who had a high Charlson comorbidity score were less likely to have stage information, while those who underwent a surgical procedure were more likely to have been staged. Among women with breast cancer, being diagnosed through an emergency admission was associated with a 24% increase in the odds of missing stage (OR = 1.24, 95%CI 1.1–1.5) compared with patients admitted by other routes. The odds of having missing stage information were 20% lower among more deprived women with breast cancer than among more affluent women (OR = 0.8, 95%CI 0.7–0.9) (Table 2).

**Table 2:** Adjusted Odds Ratios with 95% Confidence Intervals for missing stage by patient characteristics, England, 2013

	Cancer patients											
	Colon cancer				Non-small cell lung cancer				Breast cancer			
	Model 1^a^		Model 2 ^b^		Model 1^a^		Model 2 ^b^		Model 1 ^a^		Model 2 ^b^	
	OR	95% CI	OR	95% CI	OR	95% CI	OR	95% CI	OR	95% CI	OR	95% CI
Age at diagnosis												
15–64	1		1		1		1		1		1	
65–74	0.91	(0.80–1.03)	0.78	(0.36–1.71)	0.98	(0.85–1.12)	0.88	(0.48–1.62)	1.13	(0.93–1.36)	1.05	(0.55–2.03)
75–84	1.13	(1.01–1.28)	1.61	(0.84–3.11)	1.21	(1.05–1.38)	1.10	(0.60–2.01)	1.46	(1.23–1.74)	1.85	(1.06–3.24)
85+	1.84	(1.63–2.09)	2.80	(1.43–5.49)	2.08	(1.80–2.39)	2.77	(1.48–5.19)	2.15	(1.80–2.56)	3.71	(2.11–6.49)
Sex												
Male	1		1		1		1					
Female	1.09	(1.00–1.18)	1.49	(0.97–2.29)	1.05	(0.96–1.15)	1.11	(0.75–1.63)				
Income deprivation group												
More affluent	1		1		1		1		1		1	
More deprived	0.98	(0.90–1.06)	1.07	(0.70–1.66)	0.91	(0.83–1.00)	1.06	(0.68–1.65)	0.81	(0.72–0.91)	0.92	(0.63–1.35)
Emergency presentation												
No	1		1		1		1		1		1	
Yes	0.93	(0.84–1.02)	1.01	(0.65–1.57)	0.97	(0.89–1.07)	0.50	(0.31–0.78)	1.24	(1.07–1.45)	1.23	(0.71–2.14)
Death within 30 days of diagnosis												
No	1		1		1		1		1		1	
Yes	1.63	(1.44–1.85)	1.26	(0.70–2.27)	2.62	(2.37–2.89)	4.91	(3.14–7.69)	1.87	(1.47–2.38)	2.55	(1.14–5.68)
Surgical procedure												
No	1		1		1		1		1		1	
Yes	0.44	(0.40–0.48)	0.30	(0.19–0.48)	0.68	(0.58–0.79)	0.95	(0.47–1.90)	0.23	(0.20–0.28)	0.31	(0.20–0.49)
Charlson comorbidity index												
0	1		1		1		1		1		1	
1–2	1.16	(1.05–1.28)	1.40	(0.86–2.29)	1.00	(0.91–1.11)	1.07	(0.69–1.67)	1.08	(0.95–1.24)	0.95	(0.61–1.46)
> =3	1.34	(1.17–1.53)	1.26	(0.65–2.42)	1.17	(1.03–1.32)	1.01	(0.55–1.84)	1.24	(1.05–1.46)	1.72	(0.98–3.03)

*OR* odds ratio, *95% CI* 95% confidence interval

^a^ Model including the whole sample of patients ^b^ Model including only patients in the Clinical Commissioning Groups with high stage completeness

In CCGs with high stage completeness (Model 2, point estimates mutually adjusted for all the other covariates), the association between missing stage information and older age of patients remained significant and increased in strength for all three cancers (colon: OR = 2.8, 95%CI 1.4–5.5; lung: OR = 2.8, 95%CI 1.5–5.2; breast: OR = 3.7, 95%CI 2.1–6.5). Undergoing a surgical procedure remained negatively associated with missing stage among colon and breast cancer patients. Conversely, short-term mortality almost doubled the odds of having missing stage for lung and breast cancer patients, but did not have a significant effect for colon cancer.

If the whole cohort had had the probability of missing stage seen in CCGs with high completeness (low administrative issues), an additional 64, 71 and 74% of patients with missing stage for colon, lung and breast cancer, respectively, could potentially have been staged in 2013 in England (Table 3). That equates to over 10,000 out  of 95,000 patients diagnosed with one of these cancers.

**Table 3:** Number (%) of observed, expected and potentially avoidable patients with missing stage information, England, 2013

		Patients with missing information on stage at diagnosis							
		Observed		Expected ^a^			Potentially avoidable ^a^		
	Patients	No.	%	No.	95% CI	%	No.	95% CI	%
Colon cancer	21,522	3990	18.5	1433	578.7–2287.6	6.7	2557	470.2–1838.0	64.1
Non-small cell lung cancer	31,188	3935	12.6	1154	470.2–1838.0	3.7	2781	2096.9–3464.8	70.7
Breast cancer	41,657	6515	15.6	1689	838.2–2539.7	4.1	4826	3975.3–5676.8	74.1

*95% CI* 95% confidence interval

^a^ The expected number are those patients for whom stage would still be missing if all patients had had the same probability of being staged as the patients in the CCGs with the highest stage completeness. The difference between the observed and expected numbers is then the number of patients for whom missing data on stage was potentially avoidable. Numbers are rounded up to the integer

**Fig. 2:**
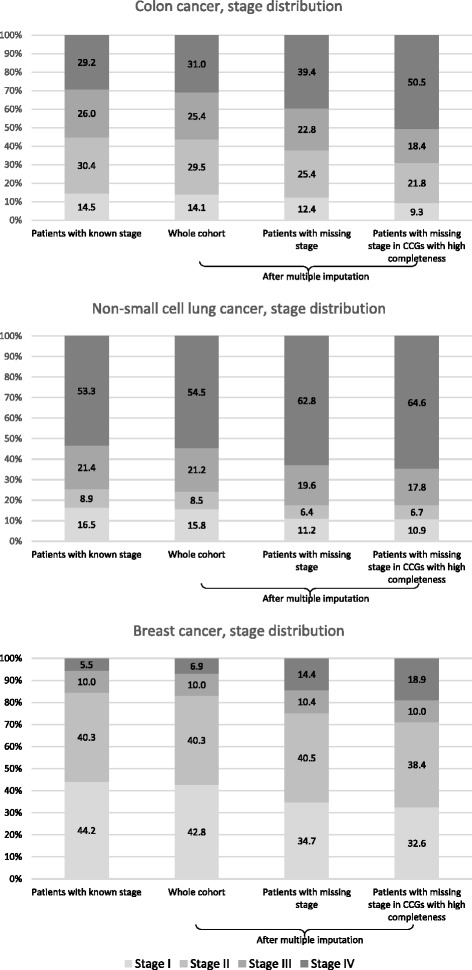
Registry stage distribution in patients with known stage, and after multiple imputation, England, 2013. Note: percentages after multiple imputation refer to the proportional distribution across all of the imputed datasets

Figure 2 shows the stage distribution under different scenarios, before and after multiple imputation. For each cancer, the stage distribution after imputation among patients with missing stage (third bar) was slightly more advanced (lower average proportion of cases in stage I and higher proportion of cases in stage IV) in comparison to the stage distribution for patients with known stage (first bar), and to the stage distribution after imputation of all patients in the cohort (second bar). The imputed stage distribution for patients with missing stage tended towards even later stages among the patients in the CCGs with high completeness (fourth bar), especially for colon and to a lesser extent for lung and breast cancer.

## Discussion

The proportion of missing stage information for colon, lung and breast cancer patients diagnosed in England in 2013 varied by socio-demographic and clinical characteristics and by geography. Multivariable modelling showed that old age was associated with missing stage irrespective of the cancer site and independent of comorbidity score, short-term mortality and patient characteristics. This remained true for patients in the CCGs with high completeness, where administrative issues were likely to be minimal. Approximately 70% of the missing data was potentially avoidable if all patients had had the same chance of being staged as the patients in those CCGs.

A strength of this study is that it draws upon a population-based, high-quality cancer registration system that covers the entire English population, minimising the risk of selection bias. Furthermore, we were able to control for receipt of surgery and for comorbidity through linkage with secondary care data (HES) data. The latter is particularly relevant for elderly patients as it allows to distinguish between clinical decisions based on the presence of concurrent chronic conditions (that may contraindicate staging investigations), and decisions based solely on chronological age. Nonetheless, we acknowledge that the Charlson Comorbidity Index only captures the clinical dimension of the frailty in the elderly [31] and does not encompass other factors (e.g. level of disability or of health care needs) which may also contribute to the absence of tumour stage.

The lack of publicly-available information on how NCRAS handled missing individual T, N, and M components in deriving the grouped stage variable used in the analysis limited some of our interpretation. The process of defining the grouped stage variable has a crucial impact on the definition of stage categories and on stage-specific survival [32]. We assumed that the derived stage reflected the true extent of the disease, that a systematic and consistent approach was used to summarise individual information on the extent of the tumour into a single variable, and that the staging classification for specific cancer sites was consistent between hospitals. However, we could not appraise directly the possibility of misclassification or inconsistencies.

The independent association between old age and missing stage that was evident in our study, has also been extensively documented by US studies using the Surveillance, Epidemiology, and End Results database, where completeness has been reported to be generally high [5, 13], and in European studies based on national or regional cancer registries with lower levels of completeness (for example, around 70% among colon cancer patients in Denmark [9] and around 50% among lung and colon patients in Mallorca [8]). Having accounted for comorbidity in our study, this result may be partially explained by the fact that elderly patients are more likely to be cared for in institutions and, in such settings, there may be sub-optimal provision of and access to cancer care which could interfere with the comprehensiveness of diagnostic and staging investigations [11]. However, a study carried out in the United States [10] reported that older patients were less likely to have missing stage regardless of the level of care needs, measured by use of home health care and nursing home care. Our finding implies inequalities in comprehensive cancer care for the elderly, which is a growing concern in the England [33].

We noted a consistent association between short-term mortality and missing stage for lung and breast cancer patients. Patients with poor prognosis may die before any diagnostic investigation can be planned and/or conducted or they may be considered to be too frail to undergo staging investigations which require a surgical procedure in many cases. In disentangling the relationship between receipt of surgical procedures and a reduced likelihood of missing stage for colon and breast cancer patients, an issue of reverse causality arises because some of these procedures had a diagnostic purpose and those with a therapeutic purpose would have been guided by stage at diagnosis.

After examining different cut-offs, we defined the threshold of high completeness at the 10th percentile of the distribution of missing stage across all CCGs because it assured both a reasonable sample size and a better than average level of completeness. This cut-off is similar to the level of completeness reached at national level in the most recent years so the results we obtained from our restricted analyses remain valid for the whole country [34]. The substantial gain in stage completeness we estimated at national level (nearly 10% for the studied cancers) represented the avoidable share of missing stage data had all CCGs performed as well as those with the most efficient registration practices in their corresponding hospital trusts in 2013. This also suggests that the administrative issues can be feasibly overcome and that strengthening registration practices (e.g. improvement of the IT systems at hospitals and registries, assignment of data liaison teams and standardisation of procedures) has been effective in enhancing the stage data completeness.

In our cohort of cancer patients, missing information followed a pattern, whereby the probability of missing stage was associated with certain socio-demographic and clinical characteristics of the patients. In addition, we observed that survival of patients with missing stage was between the survival of patients with stage III and IV tumours, and nearer to those diagnosed at stage III for breast cancer, implying a mixture of stages and not only late stage. In other words, at national level, missing stage was plausibly missing at random. In such a situation, multiple imputation represents a valid approach to handle missing data in the analytical phase [35].

However, multiple imputation is computationally intense and theoretically challenging therefore it may not be extensively applied by data analysts. Alternative techniques, such as single imputation or complete case analysis are often used, especially when easy-to-interpret routine statistics are needed in a timely and recurring fashion. Yet, depending on the reasons for the missing data, such or similar approaches may introduce bias or lead to inefficient analyses [36]. For example in England, the “missing-is-late” assumption underpins the computation of the ‘cancer diagnosed at early stage’ indicator by CCG [37] which is reported quarterly in the cancer dashboard, an online collection of cancer indicators designed to support performance monitoring and improvement [38]. According to the likely distribution we observed in our data, this strategy, which corresponds to single imputation, may be a fairer approximation in those CCGs with high completeness, where the stage distribution was more skewed towards late stages, but it may distort the results in the CCGs with lower completeness (survival is likely to be inflated because those with missing information on stage who fall into the late stage category are less likely to have late stage, in fact, than those assigned to late stage in CCGs with high completeness). Therefore, evaluating the ‘cancer diagnosed at early stage’ indicator together with a second indicator reported on the dashboard, the ‘record of cancer stage at diagnosis’, is necessary to demonstrate the extent of the potential bias and enable correct interpretations.

## Conclusions

Analyses of English population-based cancer registry data from 2013 showed that the disadvantage in stage completeness for the oldest patients persists even when completeness is generally high. This does not appear to be well determined by the presence of comorbidity. It may be due to other issues of frailty that we were unable to measure, or due to clinical decisions about diagnostic procedures being based solely on the patient’s chronological age. Given that survival of the oldest cancer patients tends to be worse in England than in other countries [1, 39], the lack of stage data should be examined in relation to poorer outcomes among the elderly.

In 2013 there remained a substantial proportion of ‘avoidable’ missing data: we estimated that 70% of patients with missing information on stage could have had complete data if administrative practices were improved to the level of the highest performing areas. These improvements have been borne out in recent years, but researchers are still using earlier data especially where they are looking at longer-term follow-up and therefore they should be aware what the characteristics of patients with missing information on stage are.

The non-random distribution of missing stage across population strata and health geographies means that handling missing stage in the analysis of population-based cancer registry data sets needs careful consideration to reduce the risk of bias. Possible reasons for missing data on stage should be carefully assessed before any study, and potential bias and distortions introduced by how missing stage is handled should be considered and acknowledged. In this way, the most appropriate analyses can be chosen and the best inferences drawn from available statistics.

## Abbreviations

CAS: Cancer Analysis System
CCG: Clinical Commissioning Group
CCI: Charlson Comorbidity Index
HES: Hospital Episode Statistics
ICD: International Classification of Diseases
ICSS: International Cancer Survival Standards
LSOA: Lower layer Super Output Areas
MAR: Missing at random
MCAR: Missing completely at random
MNAR: Missing not at random
NCRAS: National Cancer Registration and Analysis Service
NHS: National Health Service
RtD: Routes to diagnosis
TNM: Tumour Node Metastasis
UICC: International Union for Cancer Control
